# *Antheraea pernyi* Silk Fibroin-Coated PEI/DNA Complexes for Targeted Gene Delivery in HEK 293 and HCT 116 Cells

**DOI:** 10.3390/ijms15057049

**Published:** 2014-04-25

**Authors:** Yu Liu, Renchuan You, Guiyang Liu, Xiufang Li, Weihua Sheng, Jicheng Yang, Mingzhong Li

**Affiliations:** 1 National Engineering Laboratory for Modern Silk, College of Textile and Clothing Engineering, Soochow University, Suzhou 215123, China; E-Mails: liuyu@suda.edu.cn (Y.L.); yourenchuan1182@126.com (R.Y.); liuguiyang2003@163.com (G.L.); shmilysuda@126.com (X.L.); 2 School of Biology and Basic Medical Sciences, Soochow University, Suzhou 215123, China; E-Mails: shengweihua@163.com (W.S.); jcyang@suda.edu.cn (J.Y.)

**Keywords:** *Antheraea pernyi* silk fibroin, PEI (polyethylenimine), gene transfection

## Abstract

Polyethylenimine (PEI) has attracted much attention as a DNA condenser, but its toxicity and non-specific targeting limit its potential. To overcome these limitations, *Antheraea pernyi* silk fibroin (ASF), a natural protein rich in arginyl-glycyl-aspartic acid (RGD) peptides that contains negative surface charges in a neutral aqueous solution, was used to coat PEI/DNA complexes to form ASF/PEI/DNA ternary complexes. Coating these complexes with ASF caused fewer surface charges and greater size compared with the PEI/DNA complexes alone. *In vitro* transfection studies revealed that incorporation of ASF led to greater transfection efficiencies in both HEK (human embryonic kidney) 293 and HCT (human colorectal carcinoma) 116 cells, albeit with less electrostatic binding affinity for the cells. Moreover, the transfection efficiency in the HCT 116 cells was higher than that in the HEK 293 cells under the same conditions, which may be due to the target bonding affinity of the RGD peptides in ASF for integrins on the HCT 116 cell surface. This result indicated that the RGD binding affinity in ASF for integrins can enhance the specific targeting affinity to compensate for the reduction in electrostatic binding between ASF-coated PEI carriers and cells. Cell viability measurements showed higher cell viability after transfection of ASF/PEI/DNA ternary complexes than after transfection of PEI/DNA binary complexes alone. Lactate dehydrogenase (LDH) release studies further confirmed the improvement in the targeting effect of ASF/PEI/DNA ternary complexes to cells. These results suggest that ASF-coated PEI is a preferred transfection reagent and useful for improving both the transfection efficiency and cell viability of PEI-based nonviral vectors.

## 1. Introduction

The curative effect of gene therapy greatly depends on the availability of suitable gene delivery vectors. Viral gene delivery vectors are highly efficient but suffer from both immunogenicity and cytotoxicity [1,2]. Nonviral gene delivery vectors have received increasingly more attention because they exhibit both low toxicity and low immunogenicity [3,4]. Polyethylenimine (PEI), which is one of the most effective nonviral gene delivery polymers due to its proton sponge effect [5], still exhibits problems, such as toxicity, non-specificity and non-biodegradability [6–8]. Moreover, both specific targeting cells and non-specific targeting cells can endocytose positively charged particles via electrostatic interactions *in vitro*, whereas *in vivo*, to function as suitable gene carriers, the specific uptake efficiency must increase relative to the non-specific uptake efficiency [9]. Several strategies have been explored to address the shortcomings of PEI-based transfection vectors, including the construction of a new transfection system to reduce its cytotoxicity and the synthesis of novel derivatives by coupling cell targeting ligands to increase specific interactions with cells [10,11]. However, conjugating the targeting ligands directly onto PEI would likely be more complicated and may result in invalid ligand function [12,13]. Thus, it is quite desirable to seek a simpler method for PEI target gene delivery.

Silk proteins have been used successfully in the biomedical field for decades due to their excellent biocompatibility and biodegradability [14]. *Antheraea pernyi* silk fibroin (ASF), one of the most familiar species among wild silkworms, is rich in alkaline amino acids (Arg and His) and arginyl-glycyl-aspartic (RGD) tripeptide sequences [15,16]. These RGD sequences are known to be the receptors of cell integrins and to mediate special interactions between mammalian cells and extracellular matrices [17,18]. It had been reported that ASF provided much stronger cell adhesion compared to *Bombyx mori* silk fibroin (BSF) and collagen [19–22].

In our present work, a targeting system was designed not by covalently linking RGD peptides to PEI gene carriers but by electrostatically coating the PEI/DNA complexes with RGD-rich ASF to reduce cytotoxicity and improve transfection efficiency. HCT 116 cells have been reported to express abundant α_v_β_3_ and α_v_β_5_ integrins, but HEK 293 cells have been reported to possess no α_v_β_3_ and only a few α_v_β_5_ integrins [14,23]. Thus, the transfection experiments were carried out in HEK 293 and HCT 116 cells to evaluate and compare the enhanced effect of ASF-coated PEI nonviral vectors on gene transfection. For these studies, plasmid DNA encoding green fluorescent protein (GFP) was used as a reporter gene.

## 2. Results and Discussion

### 2.1. Formation of ASF (Antheraea pernyi Silk Fibroin)/PEI (Polyethylenimine)/DNA Complexes

**Figure 1.**
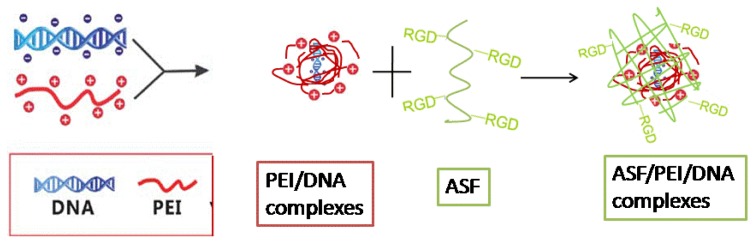
Schematic illustration of the formation of ASF (*Antheraea pernyi* silk fibroin)/PEI (polyethylenimine)/DNA ternary complexes.

In the present study, ASF/PEI/DNA complexes were designed to form nanoparticles in an aqueous solution by self-assembly. In this system, cationic PEI provided positive charges that combine with DNA to form nanoparticles, which are deposited onto the core. Then, the ASF chains were adsorbed around the nanoparticles via self-assembly to form a loop structure. Figure 1 shows a schematic illustration of the preparation of ASF/PEI/DNA complexes.

### 2.2. DNA-Binding Capability

**Figure 2.**
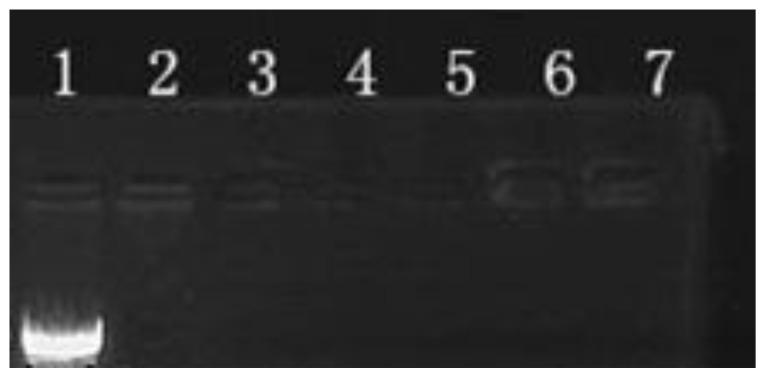
Agarose gel electrophoresis retardation assay of PEI/DNA and ASF/PEI/DNA complexes. Lane 1: naked DNA; Lanes 2–4: PEI/DNA complexes at N/P ratios of 8/1, 12/1 and 15/1, respectively; Lanes 5–7: ASF/PEI/DNA complexes at N/P ratios of 8/1, 12/1 and 15/1 by the addition of 50 μg ASF, respectively.

DNA-binding capability is a prerequisite for gene vectors [24]. The condensed form of PEI/DNA complexes can protect the DNA against enzymatic digest [25,26]. The formation of PEI/DNA and ASF/PEI/DNA complexes was examined using agarose gel electrophoresis and is shown in Figure 2. The naked DNA migrated in Lane 1 of the gel, while there was no DNA band in Lane 2, indicating that the PEI could bind DNA completely at an N/P ratio of 8/1. Further increasing the N/P ratio to 12/1 and 15/1 did not allow for the release of DNA from PEI (Lanes 3 and 4, respectively). When ASF was used to coat the PEI/DNA complex at N/P ratios of 8/1, 12/1 and 15/1 (Lanes 5 to 7, respectively), there was still no DNA migrating from the slots, indicating that ASF was attached to the complexes to form a stable ASF/PEI/DNA ternary complex that did not decompose the PEI/DNA complexes even at this weight.

### 2.3. Zeta Potential and Particle Size

The preparation of complexes is described in the experimental section, and the zeta potential and particle size of the complexes are measured and listed in Table 1. All complexes were positively charged. When the N/P ratio was 8/1, the zeta potential of the PEI/DNA complexes was 17.8 ± 0.6 mV. An increasing trend of zeta potential for PEI/DNA is observed from +17.8 to +26.6 mV with rising N/P ratios, which is attributed to the increased PEI content. The addition of negatively charged ASF to the positively charged complexes effectively decreased the surface positive potential of the PEI/DNA complexes at the same N/P ratios (approximately 1–4 mV), which should be due to the charge shielding effect of ASF on the surface of the complex. This result also indicated that ASF was deposited onto the PEI/DNA complexes, thus leading to the formation of ASF/PEI/DNA ternary complexes. Elemental analysis showed that C, N and O elements existed in the ASF/PEI/DNA_8/1_ ternary complexes (C: 75.59%, N: 11.72% and O: 12.70%). The PEI/DNA_8/1_ binary complexes packaged without ASF contained elemental C (72.61%) and N (27.39%), but no O. The appearance of elemental O also confirmed that the ASF was coated on the surface of PEI/DNA complexes, in accordance with the results of zeta potential. When the N/P ratio increased from 8/1 to 15/1, the particle size of PEI/DNA decreased from 301 to 207 nm. Compared with PEI/DNA complexes, ASF/PEI/DNA complexes possessed a slightly bigger size at equivalent N/P ratios because the ASF molecules were coated on the PEI/DNA complexes. The particle size of the ASF/PEI/DNA_8/1_ complexes was 365.3 ± 2.8 nm. With the increasing N/P ratio, the particle sizes decreased from 360 to 230 nm with a normal distribution.

**Figure 3.**
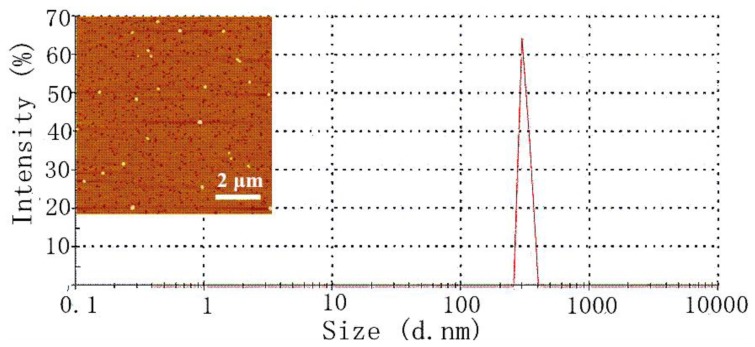
AFM height image and size distribution diagram of ASF/PEI/DNA_8/1_ ternary complexes.

Stability and dispersion are crucial to the nanoparticles for gene vector [27–29]. The AFM image indicated ASF/PEI/DNA_8/1_ ternary complexes dispersed well as spherical nanoparticles without significant aggregation. The size of ASF/PEI/DNA_8/1_ showed a relatively narrow distribution from the observation of Figure 3, in agreement with the result of the AFM analysis. So although the zeta potential deceased after coating ASF on the PEI/DNA_8/1_ complexes, the SF/PEI/DNA_8/1_ ternary complexes could maintain stable dispersion in solution. With N/P ratio growth, the ASF-coated PEI had higher positive charges to compress DNA, which was more beneficial for the stabilization of ASF/PEI/DNA ternary complexes in solution.

### 2.4. Fluorescence Microscope Observation

**Figure 4.**
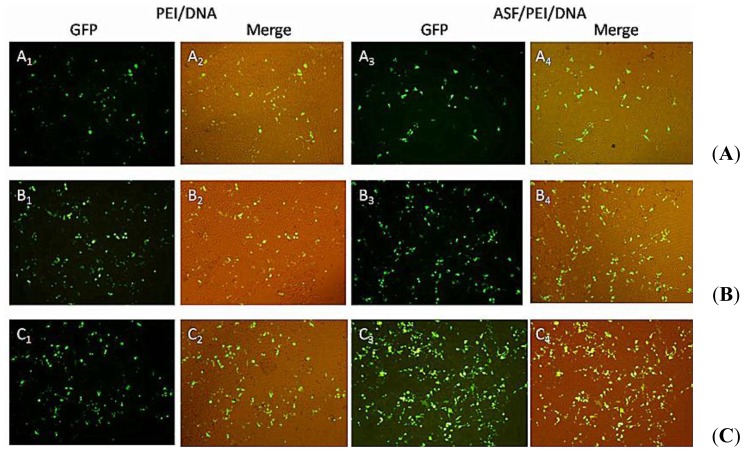
Fluorescence images of HEK 293 cells after incubation with medium containing PEI/DNA binary and ASF/PEI/DNA ternary complexes in the absence of FBS at N/P ratios of 8/1 (**A**); 12/1 (**B**) and 15/1 (**C**). After a 6 h incubation, the medium was replaced with fresh DMEM containing 10% FBS, followed by incubation for 24 h. Then the micrographs were obtained with a magnification of 100×.

**Figure 5.**
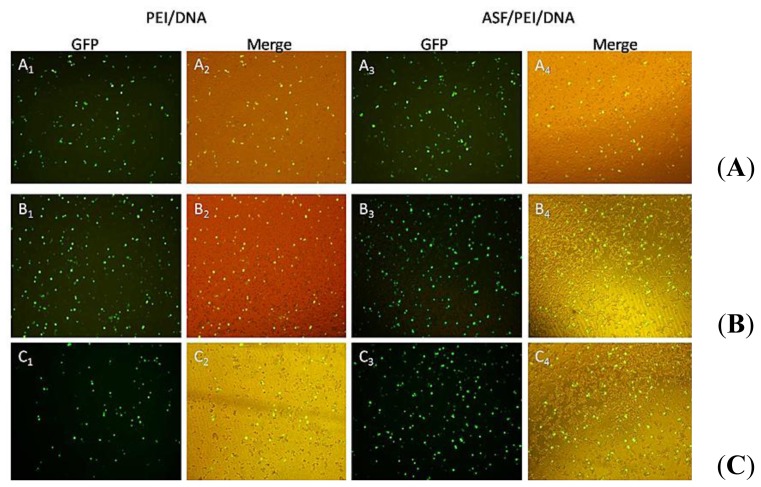
Fluorescence images of HCT 116 cells after incubation with medium containing PEI/DNA binary and ASF/PEI/DNA ternary complexes in the absence of FBS at N/P ratios of 8/1 (**A**); 12/1 (**B**) and 15/1 (**C**). After a 6 h incubation, the medium was replaced with fresh DMEM containing 10% FBS, followed by incubation for 24 h. Then the micrographs were obtained with a magnification of 100×.

Transfection experiments are carried out in HEK 293 and HCT 116 cells to evaluate and compare the enhanced targeting effect of the ASF/PEI/DNA complexes on transfection. Fluorescence micrographs of PEI/DNA binary and ASF/PEI/DNA ternary complexes in HEK 293 and HCT 116 cells are shown in Figures 4 and 5, respectively.

As shown in Figure 4, GFP expression can easily be detected from the micrographs, indicating that plasmids have been transported into the HEK 293 cells. GFP expression was fairly low 24-h post-transfection of PEI/DNA_8/1_ binary complexes (A_1_-A_2_). Moreover, the GFP expression of the ASF/PEI/DNA_8/1_ ternary complexes was no higher than that of the PEI/DNA_8/1_ binary complexes (A_3_-A_4_), which may occur because ASF shielding diminishes the electrostatic binding of the complexes to the cell surfaces, resulting in a rather lower GFP expression. Compared with PEI/DNA_8/1_ complexes, PEI/DNA_12/1_ complexes exhibited increased GFP expression (B_1_-B_2_). It was also found that only increasing the amount of PEI does not improve the GFP expression. As shown in Figure 4 (C_1_-C_2_), cells transfected by the PEI/DNA_15/1_ complexes had lower GFP expression levels than those transfected by PEI/DNA_12/1_ complexes, which may be due to the excess positive charges on PEI. ASF/PEI/DNA_15/1_ had higher GFP expression levels (C_3_-C_4_), although the presence of ASF reduced the excess positive charge of the PEI/DNA_15/1_ complexes. These results showed that PEI/DNA binary complexes without ASF had lower GFP expression levels compared to the ASF/PEI/DNA ternary complexes at high N/P ratios of 12/1 and 15/1.

Figure 5 shows the typical fluorescence micrographs of transfected HCT 116 cells. The PEI/DNA_12/1_ binary complexes exhibited higher levels of GFP expression (B_1_-B_2_) than the PEI/DNA_8/1_ complexes (A_1_-A_2_) in HCT 116 cells. GFP expression was followed by a decrease in the number of PEI/DNA_15/1_ binary complexes (C_1_-C_2_), presumably due to excess PEI decreasing cell viability. The addition of ASF into PEI/DNA_15/1_ complexes significantly enhanced GFP expression levels in HCT 116 cells (C_3_-C_4_), indicating that the RGD peptides affected the ASF binding affinity to integrins on HCT 116 cells and could compensate for the reduced electrostatic binding caused by ASF shielding as well as enhance the targeting binding affinity to the cells. Of the three N/P ratios tested in the transfection systems, ASF/PEI/DNA_12/1_ had the highest GFP expression levels according to Figure 5 (B_3_-B_4_). Furthermore, compared with GFP expression in HEK 293 cells, the intensity of GFP expression in both binary and ternary complexes in HCT 116 cells was stronger, suggesting that electrostatic interactions did not act alone during this transfection process. ASF is rich in RGD peptides, which are receptors for integrins on the cell surface; thus, ASF shielding may improve the uptake of complexes via receptors on the HCT 116 cell surfaces. Therefore, coating PEI/DNA complexes with ASF significantly improved GFP expression in HCT 116 cells. This phenomenon was further confirmed by flow cytometry results.

### 2.5. Transfection Efficiency Assay

As shown in Figures 4 and 5, GFP was successfully expressed in HEK 293 and HCT 116 cells, which means that the plasmid had been delivered into cells via ASF-coated PEI nonviral vector transfection systems. To compare the transfection efficiencies in HEK 293 and HCT 116 cells with ASF-coated PEI vectors prepared under optimal transfection conditions, the GFP expression levels were further detected quantitatively by flow cytometry.

**Figure 6.**
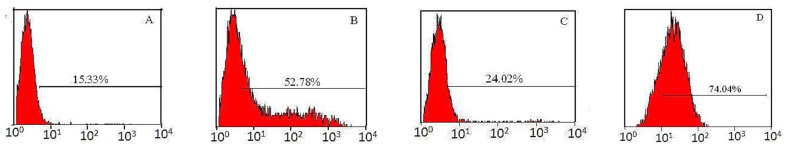
*In vitro* transfection efficiency of HEK 293 cells transfected by PEI/DNA binary and ASF/PEI/DNA ternary complexes. (**A**) PEI/DNA_12/1_; (**B**) ASF/PEI/DNA_12/1_ complexes and of transfected HCT 116 cells; (**C**) PEI/DNA_12/1_; and (**D**) ASF/PEI/DNA_12/1_ complexes.

From the results of flow cytometry in Figure 6, the transfection efficiency of PEI/DNA_12/1_ binary complexes in HEK 293 cells was 15.33% (A), whereas in HCT 116 cells, the efficiency was 24.02% (C). Notably, the transfection efficiency of the ASF/PEI/DNA_12/1_ ternary complexes was higher than that of the binary complexes in the HEK 293 cells (52.78%) (B), while it was still lower than that in the HCT 116 cells (74.04%) (D). On the one hand, unlike the excess positive charges of PEI/DNA_12/1_, the relatively weaker positive charges of ASF/PEI/DNA_12/1_ complexes, which reduced the cytotoxicity of the complexes, resulted in the enhancement of transfection efficiency. On the other hand, the RGD acid sequences, which are known to selectively recognize and bind α_v_β_3_ and α_v_β_5_ integrins, are expressed on cell surfaces [18]. The α_v_β_3_ and α_v_β_5_ integrins are expressed in normal smooth muscle cells, fibroblasts and a variety of tumor cells. Expression was low on the surface of mature vascular endothelial cells but was significantly increased in angiogenesis of endothelial cells in cancerous lesions, inflammation and wounds [30–32]. HCT 116 cells have previously been reported to express abundant α_v_β_3_ and α_v_β_5_ integrins, but HEK 293 cells have been used extensively as a gene expression tool and have been reported to possess no α_v_β_3_ and a few α_v_β_5_ integrins. Thus, RGD-rich ASF was a promoter for gene vectors and significantly enhanced the transfection efficiency in the HCT 116 cells. The transfection efficiency in HEK 293 cells was also promoted by the ASF-coated PEI nonviral vector but was still lower than that in HCT 116 cells, which may be due to differences in cell membrane integrins between HEK 293 and HCT 116 cells. These results showed that the addition of ASF could facilitate vector entry into cells through targeted uptake.

### 2.6. Cytotoxicity Study

**Figure 7.**
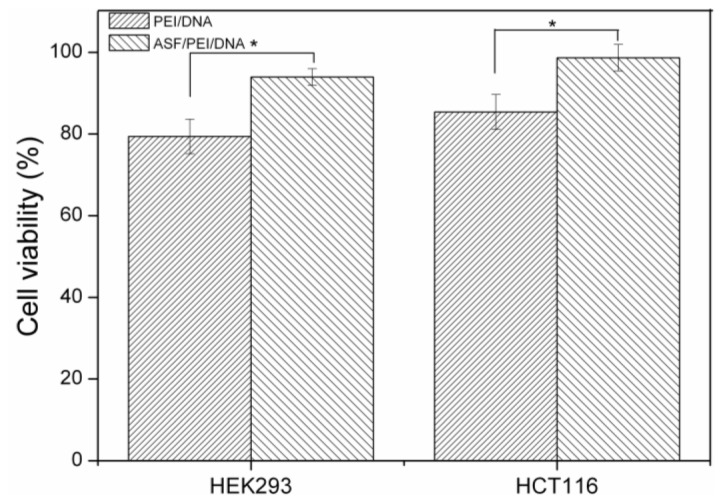
Cell viability of HEK 293 and HCT 116 cells exposed to PEI/DNA_12/1_ binary and ASF/PEI/DNA_12/1_ ternary complexes after 24 h post-transfection. * Significant difference between two groups were at *p* < 0.05.

After the composition and preparation of complexes, which were the same as those used in the transfection efficiency experiments, the cytotoxicity of the complexes was measured using a CCK-8 assay. It was shown in Figure 7 that approximately 93% of the HEK 293 cells were alive after treatment with ASF/PEI/DNA_12/1_ complexes, whereas only approximately 80% of the cells were alive after treatment with PEI/DNA_12/1_ complexes. Unlike the excess positive charges of PEI/DNA_12/1_, the ASF/PEI/DNA_12/1_ complexes had relatively weaker positive charges, which reduced the cytotoxicity of the complexes. These results were also confirmed by the high viability (greater than 96%) of the HCT 116 cells transfected with ASF-coated PEI carriers, thus demonstrating that the presence of ASF in the formulations could reduce both the surface charge and cytotoxicity.

### 2.7. LDH Release Assays

The generally accepted transfection mechanism of nonviral gene carriers is a three-step process that includes binding to the cell membrane, internalization into cells, and escaping from the endosome. It has been reported that in the transfection process, the internalization of complexes could induce the formation of transient, nanoscale holes in living cells and that these holes would allow for a greatly enhanced exchange of nanoparticles across the cell membrane [9]. Thus, lactate dehydrogenase (LDH) would be released from these holes. In this work, the interaction of PEI/DNA_12/1_ and ASF/PEI/DNA_12/1_ complexes with HEK 293 and HCT 116 cells was evaluated by measuring the release of LDH in the transfection process.

**Figure 8.**
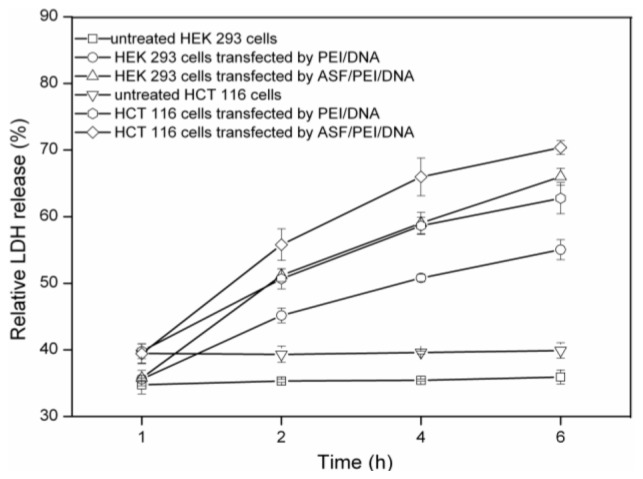
LDH release profiles of HEK 293 and HCT 116 cells exposed to PEI/DNA_12/1_ binary and ASF/PEI/DNA_12/1_ ternary complexes during the incubation for 6 h.

Figure 8 shows the relative LDH release profiles at 1, 2, 4 and 6 h for untreated HEK 293 and HCT 116 cells, as well as for those cells transfected by PEI/DNA_12/1_ and ASF/PEI/DNA_12/1_ complexes. At 1 h after exposing the cells to the complexes, the amounts of LDH released from the cells were nearly the same. None of the untreated cells released a significantly increased amount of LDH over time, but HEK 293 cells released less LDH than HCT 116 cells. After exposure to PEI/DNA_12/1_ complexes, HEK 293 cells released more LDH than the untreated cells after 1 h. This result was due to the positive charges of the PEI/DNA_12/1_ complexes, which caused LDH release while crossing the cell membrane. For the transfected HEK 293 cells, the amount of LDH released increased with time prolonged, indicating that increasingly more complexes were endocytosed by the cells. Meanwhile, the ASF/PEI/DNA_12/1_ complexes caused more LDH release from 1 to 6 h than did PEI/DNA_12/1_ in HEK 293 cells, demonstrating that more ASF/PEI/DNA_12/1_ complexes were endocytosed by cells than PEI/DNA_12/1_ complexes. In addition, LDH released in HCT 116 cells followed the same trend observed in HEK 293 cells but was more extensive under the same conditions. This result was in good agreement with the transfection efficiency trend shown in Figure 7, demonstrating that the transfection efficiency of all complexes in HCT 116 cells was better than that in HEK 293 cells. This difference may be attributed to the different targeting binding affinity for cells of ASF/PEI/DNA_12/1_ and PEI/DNA_12/1_. The ASF coating on PEI/DNA complexes may enhance the binding affinity for the cells to compensate for the reduced electrostatic binding affinity for cells, thus facilitating internalization into cells and finally improving the transfection efficiency.

The present study constructed ASF-coated PEI nonviral transfection systems via an encapsulation method. ASF is characterized by RGD tripeptide sequence and is reported to have much stronger cell adhesion capacity [33]. Thus, we anticipate improving our system by using an ASF coating to reduce cytotoxicity and further enhance the transfection efficiency in both HEK 293 and HCT 116 cells. Here, the transfection efficiency and cytotoxicity have been analyzed as a function of the ratio of PEI to DNA, ASF content and cell type. The complexes were prepared at N/P ratios of 8/1, 12/1, and 15/1, with an ASF coating of 50 μg. After incubating for 45 min to obtain complexes homogeneous in size, gel electrophoresis results showed that PEI could completely bind DNA. The highest transfection efficiency in HEK 293 cells occurred with an ASF-coated PEI gene vector and was shown to be nearly 52.78%. Moreover, this transfection system exhibited greater efficiency in HCT 116 cells, achieving up to 74.04%, which means that the ASF-coated PEI gene vector promotes transfection more easily in HCT 116 cells than in HEK 293 cells. It has previously been reported that RGD acid sequences could selectively recognize and bind both α_v_β_3_ and α_v_β_5_ integrins on the membrane surfaces of certain cell types. Therefore, relatively higher levels of transfection efficiency were achieved by using ASF-coated PEI in cells, especially in cells that had integrins on their cell membranes. Meanwhile, a large number of viable cells were found after transfection with the ASF-coated PEI system in both HEK 293 and HCT 116 cells. In contrast, lower cell viability with PEI/DNA_12/1_ complexes was observed after transfection. These results demonstrate that the presence of ASF significantly decreased the cytotoxicity.

## 3. Materials and Methods

### 3.1. Materials

PEI (branched, M.W. 25000) was purchased from Sigma Aldrich (St. Louis, MO, USA). Dulbecco’s modified eagle medium (DMEM), fetal bovine serum (FBS) and 0.25% trypsin-EDTA were obtained from Invitrogen Corp. (Carlsbad, CA, USA). A cell counting kit-8 was obtained from Dojindo Corp. (Rockville, MD, USA). A lactate dehydrogenase (LDH) release assay kit was obtained from Beyotime Institute of Biotechnology (Nantong, China). Plastic cell culture dishes, plates and flasks were obtained from Corning Corp. (Lowell, MA, USA). The main instruments used include an inverted fluorescence microscope (Olympus IX71, Olympus, Tokyo, Japan), microplate reader (Sunrise Tecan, Sydney, Australia) and flow cytometer (FACS, San Jose, CA, USA).

### 3.2. Cell Culture

Human embryonic kidney (HEK 293) cells and human colorectal carcinoma (HCT 116) cells were purchased from the Shanghai Cell Center (Shanghai, China). These cells were cultured in Dulbecco’s modified eagle medium (DMEM) with 10% fetal bovine serum (FBS) at 37 °C in a humidified atmosphere with 5% CO_2_.

### 3.3. Plasmid DNA Production

Plasmid DNA encoding GFP was obtained from the Laboratory of Molecular Biology of Soochow University and was propagated in competent Escherichia coli DH5a cells (Invitrogen, Carlsbad, CA, USA). Ultrapure, endotoxin-free plasmid DNA was prepared using a QIA filter kit (Qiagen, Chatsworth, CA, USA) according to the manufacturer instructions. The plasmid concentration and its purity were measured by ultraviolet (UV) absorbance at 280 and 260 nm on a Nanodrop 2000 UV spectrophotometer (Thermo Fisher Scientific, Waltham, MA, USA).

### 3.4. Preparation of ASF Solution

As described previously [20], *Antheraea pernyi* silk (Dandong, Liaoning, China) was treated three times with 2.5 g/L Na_2_CO_3_ solution at 98–100 °C for 30 min to remove sericin. The degummed ASF fibers were dissolved in melted Ca(NO_3_)_2_·4H_2_O solutions at a 1:10 (*w*/*v*) bath ratio for 5 h at 105 °C. The mixed solution was then dialyzed against distilled water for 96 h at 25 ± 0.5 °C with a cellulose tube (M.W. 9000, Pierce, Rockford, IL, USA) to remove excess salt. The fresh solution was then filtered and degassed using a 0.22 μm pore size filter.

### 3.5. Preparation of ASF-Coated PEI Complexes

The PEI solution was adjusted to 1 mg/mL with deionized water and was passed through a 0.22 μm pore size filter prior to mixing with DNA solution. The complexes were prepared by vortexing different volumes of the PEI solution with DNA solution at N/P ratios of 8/1, 12/1 and 15/1 followed by incubation for 45 min at room temperature. After PEI/DNA suspension was formed, 50 μg of ASF solution was mixed with the PEI/DNA complexes. The mixture was briefly vortexed and incubated for 45 min to form the ASF/PEI/DNA complexes.

### 3.6. Gel Retardation Assay

A gel retardation assay was used to determine the DNA condensation ability of PEI. The PEI/DNA and ASF/PEI/DNA complexes in Milli-Q water were prepared as described in the section above and were then injected into 1.0% agarose gel containing 0.5 μg/mL ethidium bromide. Each well of the gels was loaded with complexes containing 1 μg of plasmid DNA. After loading the complexes onto the agarose gel, electrophoresis was carried out in constant voltage mode at 80 V for 20 min.

### 3.7. Zeta Potential, Particle Size Measurements and Elemental Analysis

Sizes of the PEI/DNA and ASF/PEI/DNA complexes were measured using a Zetasizer Nano ZS90 (Malvern, UK). Briefly, 1 mL of freshly prepared sample solution was suspended in filtered deionized water and sonicated to obtain uniform dispersion of nanoparticles. The data represented the average values of twenty measurements with accumulation times of 300 s. The surface charges (zeta potentials) of the PEI/DNA binary and ASF/PEI/DNA ternary complexes were also measured using the same equipment at 25 °C (*n* = 20). Energy-dispersive X-ray spectroscopy (EDS) (EDAX Inc., Mahwah, NJ, USA) was utilized for the elemental analysis. The PEI/DNA and ASF/PEI/DNA complexes samples at N/P ratios of 8/1 were loaded on a silicon wafer. After an overnight incubation to air dry, EDS was utilized to scan the mass fraction of three elements: carbon, nitrogen and oxygen.

### 3.8. In Vitro Transfection

HEK 293 and HCT 116 cells were cultured in 6-well plates at a density of 1 × 10^6^ cells per well in growth medium for 24 h prior to transfection. After 24 h of cell attachment, the transfection experiments were conducted at approximately an 80% confluence of cells. Prior to the addition of PEI/DNA binary or ASF/PEI/DNA ternary complexes, the culture medium was removed from each dish, and the cells were then washed three times with PBS. Binary and ternary complexes containing 2 μg of DNA were then diluted in 0.5 mL of DMEM and added dropwise to the cells. After a 6 h incubation, the medium was replaced with fresh DMEM containing 10% FBS, followed by incubation for 24 h. After 24-h post-transfection, fluorescent images of cells were acquired using an inverted fluorescence microscope (Olympus IX71).

### 3.9. Transfection Efficiency Evaluation

To quantify and compare the transfection efficiency of the PEI/DNA and ASF/PEI/DNA complexes, the percentages of cells expressing GFP were measured via flow cytometer. Briefly, HEK 293 and HCT 116 cells were seeded at a density of 1 × 10^6^ cells/well and incubated with PEI/DNA and ASF/PEI/DNA complexes in 6-well plates. After the desired incubation time, the cells were harvested by addition of 0.5 mL of 0.25% trypsin to the samples. The trypsinized cell suspensions were neutralized with 1 mL of complete medium, followed by centrifugation at 1000 rpm for 10 min. Cell pellets were washed twice with PBS and centrifuged for 10 min at 1000 rpm. The resultant cell samples were resuspended in 0.2 mL of PBS and analyzed via flow cytometry. For each sample, 10,000 cells were collected. The experiments were conducted in quadruplicates and repeated three times.

### 3.10. Cell Viability Analysis

The cell viability of the HEK 293 and HCT 116 cells 24 h post-transfection of the PEI/DNA and ASF/PEI/DNA complexes was evaluated by the CCK-8 method (Sigma, St. Louis, MO, USA) according to the manufacturer’s instructions [24]. Cells not exposed to the complexes were used as a control. The absorbance of each well was read on a microplate reader at 450 nm. Cell viability was evaluated by calculating the percentage of sample absorbance compared to that of the control after subtracting the blank absorbance. Each experiment was performed in triplicate and repeated a minimum of three times.

### 3.11. Complexes Interactions with Cell Membranes

Interactions of the complexes with cell membranes were evaluated by measure LDH release [25]. According to an LDH release experiment [9], HEK 293 and HCT 116 cells were seeded into 6-well plate at 1.0 × 10^6^/well. After a 24-h incubation time, primary DMEM was aspirated from each well and replaced with 1 mL of serum-free DMEM. Each 6-well plate was divided into two parts of maximum LDH and experimental LDH release groups. Solutions of PEI/DNA and ASF/PEI/DNA complexes were added to experimental LDH release samples and incubated for 1–6 h. Next, 100 μL of LDH lysis solution was added to the maximum LDH release sample wells and was incubated for 1 h at 37 °C. The 6-well plates were covered with aluminum foil and shaken at room temperature for 30 min. The supernatant was centrifuged for 5 min at 400 rpm, and its absorbance was then read at 492 nm. The untreated cells were taken as blank controls. The release of relative LDH was calculated by the following equation:

(1) relative LDH release (%)=(experimental-blank)/(maximum-blank)×100

Each experiment was performed in triplicate and repeated a minimum of three times.

### 3.12. Statistical Analysis

Data were expressed as the means ± standard deviation. All statistical analysis was performed with one-way ANOVA, and differences were considered statistically significant at *p* < 0.05.

## 4. Conclusions

This is the first report of transfection into HEK 293 and HCT 116 cells using ASF-coated PEI gene vectors. ASF was used to shield the PEI/DNA complexes, to reduce their cytotoxicity and to enhance gene transfection into HEK 293 and HCT 116 cells. Furthermore, based on the RGD cell-binding motifs in ASF, the ASF/PEI/DNA complexes strongly promoted enhanced transfection efficiency in HCT 116 cells, which have integrins on their surfaces. The results of LDH release were in good agreement with the transfection efficiency trend; that is, the ASF/PEI/DNA complexes caused more LDH release than did PEI/DNA, especially in the HCT 116 cells. These results suggest that this ASF-coated PEI carrier is a more facile and effective targeting vector than PEI alone, and it could be developed as a promising tool to improve the gene transfection ability of nonviral gene vectors.

## Figures and Tables

**Table 1.** The zeta potential and particle size of complexes at different N/P ratios.

Samples	N/P Ratio	Zeta Potential (mV)	Average Diameter (nm)
PEI/DNA_8/1_	8/1	17.8 ± 0.6	301.7 ± 4.6
PEI/DNA_12/1_	12/1	19.8 ± 0.6	247.8 ± 3.0
PEI/DNA_15/1_	15/1	26.6 ± 0.9	207.1 ± 5.0
ASF/PEI/DNA_8/1_	8/1	1.3 ± 0.4	365.3 ± 2.8
ASF/PEI/DNA_12/1_	12/1	2.4 ± 0.5	281.9 ± 6.9
ASF/PEI/DNA_15/1_	15/1	3.7 ± 0.5	230.6 ± 4.3

Data were expressed as the means ± standard deviation (*n* = 20).
